# The Impact of Postoperative Pulmonary Complications on Perioperative Outcomes in Patients Undergoing Pneumonectomy: A Multicenter Retrospective Cohort Study of the German Thorax Registry

**DOI:** 10.3390/jcm13010035

**Published:** 2023-12-20

**Authors:** Axel Semmelmann, Wolfgang Baar, Nadja Fellmann, Isabelle Moneke, Torsten Loop

**Affiliations:** 1 Department of Anesthesiology and Critical Care, Medical Center—University of Freiburg, Faculty of Medicine, University of Freiburg, 79106 Freiburg, Germany; 2 Department of Thoracic Surgery, Medical Center—University of Freiburg, Faculty of Medicine, University of Freiburg, 79106 Freiburg, Germany; 3 German Society of Anaesthesiology and Intensive Care Medicine, 90115 Nürnberg, Germany

**Keywords:** postoperative pulmonary complications, perioperative mortality, pneumonectomy, thoracic surgery, anesthesia

## Abstract

Postoperative pulmonary complications have a deleterious impact in regards to thoracic surgery. Pneumonectomy is associated with the highest perioperative risk in elective thoracic surgery. The data from 152 patients undergoing pneumonectomy in this multicenter retrospective study were extracted from the German Thorax Registry database and presented after univariate and multivariate statistical processing. This retrospective study investigated the incidence of postoperative pulmonary complications (PPCs) and their impact on perioperative morbidity and mortality. Patient-specific, preoperative, procedural, and postoperative risk factors for PPCs and in-hospital mortality were analyzed. A total of 32 (21%) patients exhibited one or more PPCs, and 11 (7%) died during the hospital stay. Multivariate stepwise logistic regression identified a preoperative FEV_1_ < 50% (OR 9.1, 95% CI 1.9–67), the presence of medical complications (OR 7.4, 95% CI 2.7–16.2), and an ICU stay of more than 2 days (OR 14, 95% CI 3.9–59) as independent factors associated with PPCs. PPCs (OR 13, 95% CI 3.2–52), a preoperative FEV_1_ < 60% in patients with previous pulmonary infection (OR 21, 95% CI 3.2–52), and continued postoperative mechanical ventilation (OR 8.4, 95% CI 2–34) were independent factors for in-hospital mortality. Our data emphasizes that PPCs are a significant risk factor for morbidity and mortality after pneumonectomy. Intensified perioperative care targeting the underlying risk factors and effects of PPCs, postoperative ventilation, and preoperative respiratory infections, especially in patients with reduced pulmonary reserve, could improve patient outcomes.

## 1. Introduction

Patients with extensive lung cancer or other diseases requiring who undergo pneumonectomy surgery have a high perioperative risk of morbidity and mortality [1,2].

Recent advances in perioperative care, surgical techniques, and systemic oncologic therapy have led to improved outcomes in lung cancer patients, allowing those previously considered as inoperable to receive surgical therapy [3,4].

However, short-term morbidity and mortality have not changed significantly in patients undergoing pneumonectomy who experience postoperative pulmonary complications (PPC), which are the leading cause of non-surgical morbidity. In spite of this, within the recent literature, there is little information regarding perioperative risk factors [5].

The aim of this clinical study was to identify the effect of patient and procedure-related elements, along with postoperative factors, on the incidence of postoperative pulmonary complications as the primary endpoint. In addition, the effects of surgical, pulmonary, and non-pulmonary complications on perioperative morbidity and mortality were quantified as secondary endpoints.

## 2. Materials and Methods

### 2.1. Data Source

**Figure 1:**
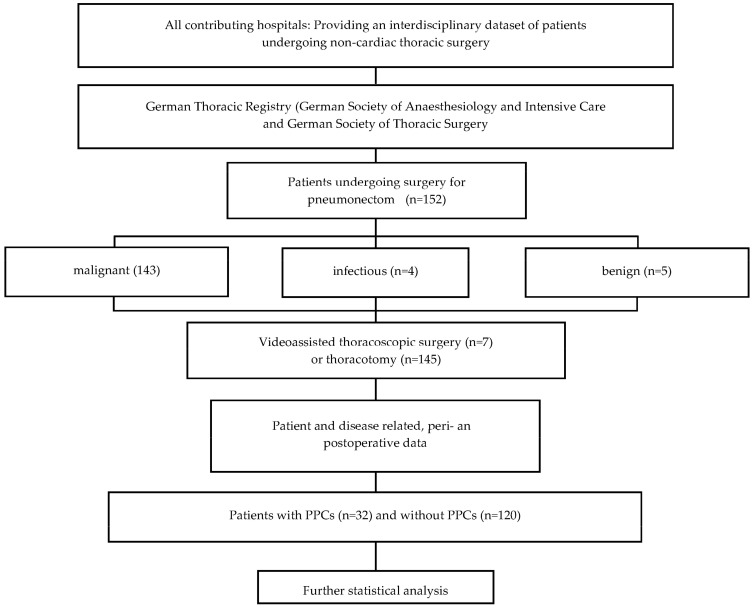
Consort diagram depicting the case selection. PPC = postoperative pulmonary complications.

The data were collected from the German Thorax Registry, a retrospective multicenter cohort register and database reporting >150 perioperative items, investigating patients undergoing noncardiac thoracic surgery from 2017–2021. A total of 152 consecutive patients undergoing pneumonectomy at all 4 contributing tertiary care hospitals (University Hospital of Freiburg, the Hospital of the University Witten/Herdecke-Cologne, and the University Hospitals of Düsseldorf and Munich) were identified during this 4-year period and were included in this analysis (Figure 1). The data were submitted via a web-based application into a central database. Patient data were anonymized.

### 2.2. Ethics

The present study was performed according to the published guidelines of the German Thorax Registry. The application for data analysis was granted by the advisory board. Ethics approval for this study (Ethical Committee of the University Witten/Herdecke, approval No.: 64/2014, 24 June 2014) was obtained.

The study was planned and designed in compliance with the initiative for strengthening the reporting of observational studies in epidemiology (STROBE) (Supplementary Table S1) [6].

### 2.3. Patient-Related Factors

Figure 1 defines the selection process for the cases presented in this study. Patient-specific risk factors included gender, body mass index (BMI), age, ASA physical status, smoking status, and the presence of a preoperative pulmonary infection within 4 weeks before surgery. The preoperative therapies were examined, including previous lung surgery, as well as neoadjuvant/preoperative radio- and/ or chemotherapy (RCT). Preoperative laboratory parameters (hemoglobin, leukocytes, C-reactive protein) and preoperative pulmonary function tests (forced expiratory volume in one second (FEV_1_), functional vital capacity (FVC), and diffusion capacity of carbon monoxide (DLCO single breath), expressed as percentages of the predicted value, and the results of the capillary blood gas analysis were analyzed.

### 2.4. Procedure-Related Factors

The extent of the surgical resection, amount of intraoperative blood loss, the duration (incision–suture time), and location of the operation were analyzed. Relevant complications requiring surgical or interventional therapy during the primary postoperative hospital stay were documented. Surgical complications such as the rate of reoperations, chylothorax, bronchial stump insufficiency (BSI), postoperative empyema, postoperative bleeding, duration of bronchopleural fistula (BPF), and subsequent need for a new chest drain were recorded. The perioperative management was planned and performed by the anesthesiologist in charge. All patients underwent the pneumonectomy under general anesthesia, with one-lung ventilation (OLV). Analysis of the anesthetic management focused on the type of regional anesthesia, i.e., continuous thoracic epidural analgesia (TEA) or continuous thoracic paravertebral block, single shot paravertebral block (PVB), or intercostal nerve block (ICB). The intraoperative ventilator settings (fraction of inspired oxygen (FiO_2_), respiratory rate per minute, level of positive endexpiratory airway pressure (PEEP), inspiratory airway pressure, and driving pressure) indicating invasiveness of ventilation and additional measures in case of hypoxemia during OLV (pulmonary artery banding, CPAP to the nonventilated lung) and the duration of OLV were recorded. The amount of intraoperative infusion of crystalloids, colloids, and blood products transfused was analyzed. The sum of blood loss and urine output was subtracted from the total volume of fluids infused, and the resulting net intraoperative balance was calculated to assess the effects on postoperative pulmonary complications.

### 2.5. Postoperative Factors

Complications with therapeutic consequences were recorded during the primary postoperative hospital stay.

The general assessment of the postoperative respiratory status included oxygen requirement, along with the respiratory parameters (respiratory rate, Horowitz oxygenation index (PaO_2_ (mmHg)/FiO_2_), ventilator settings) for spontaneous breathing and postoperative mechanical ventilation, if primary extubation in the operation theater was not possible. The need for therapeutic non-invasive ventilation (NIV) for respiratory failure, the rate of re-intubation and mechanical ventilation following the operation, and the duration of mechanical ventilation were analyzed.

Postoperative pulmonary complications (PPC) were the primary endpoint of the study due to their deleterious effects on pneumonectomy patients.

PPCs were defined as the following

- Acute respiratory distress syndrome according to the Berlin classification, excluding the criterion regarding “bilateral infiltrates” after pneumonectomy [7];
- Postoperative respiratory failure, with increasing oxygen requirement (≥4 L/min) and intensified physiotherapy;
- Re-intubation following the postoperative endotracheal extubation due to respiratory failure;
- Therapeutical non-invasive ventilation (NIV) for respiratory failure;
- New onset postoperative pneumonia (EPCO), defined as: new pulmonary infiltrates with associated leukocytosis, fever, new purulent sputum, the need for antibiotic therapy, and increased oxygen demand [8];
- The need for extracorporeal membrane oxygenation (ECMO) for respiratory failure subsequent to mechanical ventilation.

The definitions chosen in our study aimed at identifying severe complications and were tailored to the specific patient group. Commonly used PPCs, including pneumothorax and pleural effusion on the operative side, are not necessarily complications after pneumonectomy.

The secondary outcomes included the incidence of postoperative medical complications, surgical complications, postoperative in-hospital mortality during the primary postoperative stay until discharge, and the length of postoperative stay, including ICU and total hospital stay. Postoperative medical, non-respiratory complications included the incidence of cardiac complications (new onset arrhythmia, myocardial infarction, cardiac failure), renal failure, sepsis, delirium, cardiac arrest, and severe neurologic gastrointestinal complications. Surgical complications included re-operation after the primary operation, postoperative empyema, chylothorax, surgical site infection, and postoperative hematothorax.

The primary postoperative admission unit (post anesthesia care unit (PACU), intensive care unit (ICU), high dependency unit (HDU)) was recorded.

The total postoperative length of hospital stay (LOS), days in HDU/ICU, and survival during the hospital stay were calculated. If a patient was discharged from the hospital, survival was assumed, unless reported otherwise. Readmission rates and mortality after primary discharge were beyond the scope of this study.

### 2.6. Statistics

For statistical analysis, IBM SPSS Statistics for Windows^®^ (Version 23.0 Armonk, NY, USA: IBM Corp.) was used. Univariate analysis was performed by dividing the specific cohorts into two groups according to the dependent variable. Continuous variables were calculated using the Mann–Whitney U test and presented as mean ± standard deviations. Continuous and ordinal variables were dichotomized and transformed into categorical variables using a stepwise approach to determine cut-off values. Categorical variables were calculated with the *X*^2^-test. All tests were two-tailed, and a *p*-value of <0.05 was considered statistically significant. Only statistically significant parameters with a *p* < 0.005 in the univariate analysis were included in the multivariate stepwise logistic regression analysis to identify their independent statistical effects on the respective variable. Survival was computed using a Kaplan–Meier estimator, evaluating the differences using a log-rank test.

## 3. Results

**Figure 2:**
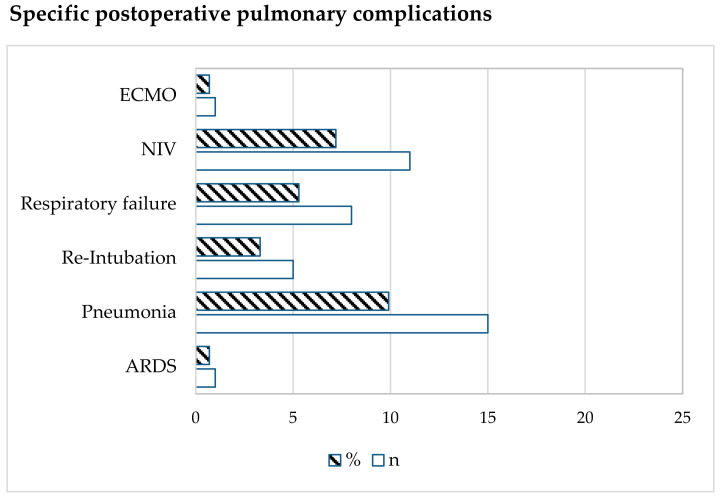
Type of postoperative pulmonary complication. Data are expressed as number (n) or %. ECMO = extracorporeal membrane oxygenation; NIV = non-invasive ventilation; ARDS = Acute Respiratory Distress Syndrome.

The incidence of one or more PPCs after pneumonectomy in this group was 21% (32/152). A total of 19 patients exhibited one PPC, 7 patients had 2 PPCs, and 6 patients presented 3 or more PPCs. The specific PPCs are shown in Figure 2.

**Figure 3:**
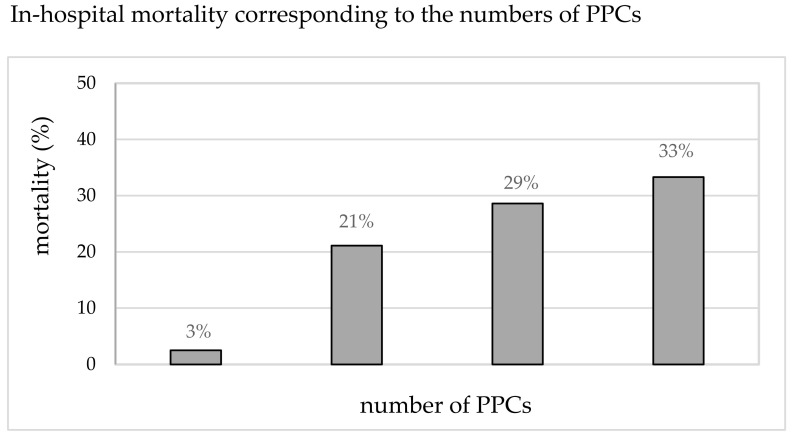
In-hospital mortality (%) corresponding to the number of PPCs.

Table 1 shows the results of the univariate analysis comparing the patient-specific and preoperative characteristics of patients undergoing pneumonectomy, with or without PPCs. The procedure-related risk factors (anesthesiologic and surgical) are presented in Table 2. The association between the incidence of PPCs and in-hospital mortality is illustrated in Figure 3. Compared to the mean mortality of 7% (11/152), the mortality in patients with PPCs was significantly increased and correlated with the number of PPCs (3%, 21%, 29%, and 33% for 0, 1, 2, and ≥3 PPCs). The total mortality in patients with any PPC was 25% (8/32), compared to that in patients without PPCs (*p* < 0.001).

After the univariate analysis, preoperative factors, including a FEV_1_ < 50% (7/32 vs. 9/120, *p* < 0.001) or a recent (<4 weeks preoperatively) pulmonary infection with a FEV_1_ < 60% (3/32 vs. 4/120, *p* = 0.04), were significantly associated with the increased incidence of PPCs. Among the procedural factors, a longer incision to suture time (178 ± 68 vs. 221 ± 98 min, *p* < 0.006) was observed in patients with PPCs. Anesthesiological factors, such as a lower FiO_2_ during OLV (0.61 ± 0.11 vs. 0.69 ± 0.15, *p* = 0.015), a lower inspiratory pressure (20.4 ± 4.3 vs. 23.2 ± 3.9 mbar, *p* = 0.036), and a lower driving pressure during OLV (14.1 ± 4.2 vs. 17.1 ± 3.9 mbar, *p* = 0.03) were associated with a lower rate of PPCs.

Postoperative differences between patients with and without PPCs are shown in Table 3. Continued postoperative mechanical ventilation was more frequent (6/32 vs. 7/120, *p* = 0.03) and of longer duration (2.6 ± 8 vs. 140 ± 223 h, *p* < 0.001), as were ICU (1.94 ± 1.4 vs. 10.5 ± 12.5 days, *p* < 0.001), and hospital stays (10.8 + 5 vs. 21.4 + 19.8 days, *p* < 0.001) in patients with PPCs. Patients with PPCs had significantly more medical (15/32 vs. 14/120, *p* < 0.001) and surgical (13/32 vs. 15/120, *p* = 0.003) complications during the postoperative period.

**Figure 4:**
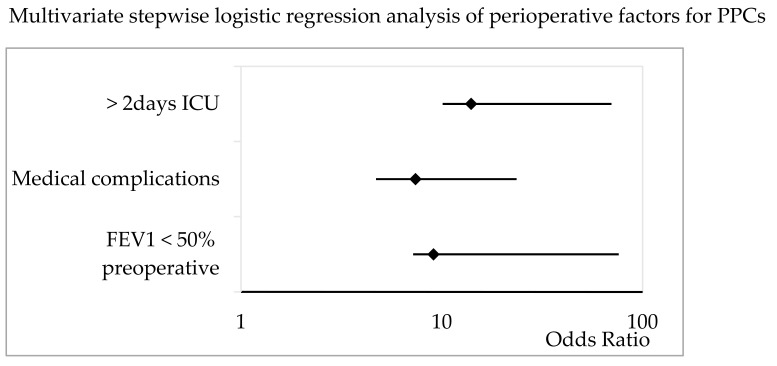
Multivariate stepwise logistic regression analysis of patient-specific, procedural, and postoperative factors for PPCs in patients undergoing pneumonectomy. The Odds Ratio and 95% CI are shown. ICU = intensive care unit; FEV_1_ = forced expiratory volume in 1 s.

The multivariate, stepwise logistic regression analysis demonstrated a preoperative FEV_1_ < 50% (OR 9.1, 95% CI 1.9–67), medical complications (OR 7.4, 95% CI 2.7–16.2), and an ICU stay of more than 2 days (OR 14, 95% 3.9–59) to be independent factors associated with the incidence of PPCs (Figure 4).

Table 4 presents the results from the univariate analysis of patient-related and preoperative risk factors for in-hospital mortality: a respiratory infection within 4 weeks before the operation (4/11 vs. 18/141, *p* = 0.04), a lower preoperative FEV_1_ (58 ± 25.4 vs. 73 ± 20% predicted, *p* = 0.037), and a FEV_1_ < 60%, combined with a previous pulmonary infection (4/11 vs. 4/141, *p* < 0.001). Furthermore, higher percentages of neoadjuvant radiation (4/11 vs. 6/141, *p* = 0.005) and chemotherapy (5/11 vs. 16/141, *p* = 0.02) were identified in non-survivors.

Table 5 presents the differences in anesthesiologic and surgical factors. An OLV duration > 180 min in both patients with a predicted DLCO < 60% (3/11 vs. 9/141, *p* = 0.04), and a predicted FEV_1_ < 60% (3/11 vs. 9/141, *p* = 0.04), an increased intraoperative crystalloid infusion (3537 ± 2925 vs. 2406 ± 1304 mL, *p* = 0.015), higher blood loss (2059 ± 2493 vs. 631 ± 545 mL, *p* < 0.001), and a higher transfusion rate were observed in non-survivors. The duration of surgery (221 ± 98 vs. 178 ± 68 min, *p* = 0.006), the duration of the presence of the chest tube (14.5 ± 12 vs. 2.2 ± 3.5 d, *p* < 0.001), and the frequency of re-operations (4/11 vs. 14/141, *p* = 0.027) varied significantly between non-survivors and survivors.

**Figure 5:**
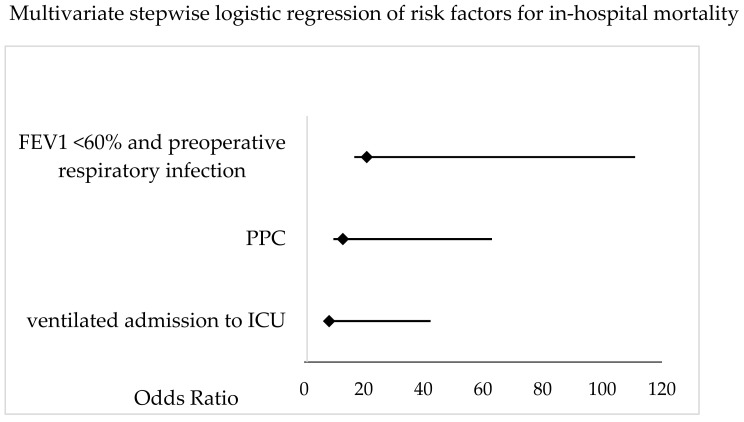
Multivariate stepwise logistic regression analysis of patient-specific, procedural and postoperative factors for postoperative, in-hospital mortality in patients undergoing pneumonectomy. The Odds Ratio and 95% CI are shown. FEV_1_ = forced expiratory volume in 1 s; PPC = postoperative pulmonary complication; ICU = intensive care unit.

Postoperative differences are shown in Table 6. Additional data is provided in the Appendix A covering medical complications (Table A1, Table A2, Table A3, Table A4, Table A5 and Table A6). Non-survivors had longer ICU (16.8 ± 22 vs. 3.6 ± 6 days, *p* > 0.001) and hospital (21.4 ± 19.8 vs. 10.8 ± 5 days, *p* < 0.001) stays, as well as higher rates (4/11 vs. 9/141, *p* = 0.008) and longer durations of postoperative ventilation (302 ± 467 vs. 28 ± 105 h, *p* < 0.001). Non-survivors exhibited significantly more postoperative complications, including PPCs (8/11 vs. 24/141, *p* < 0.001), medical complications (6/11 vs. 23/141, *p* < 0.001), and surgical complications (6/11 vs. 25/141, *p* = 0.01). The multivariate, stepwise logistic regression analysis identified a preoperative FEV_1_ < 60% predicted in patients with a preoperative pulmonary infection (OR 21, 95% CI 4.2–103, *p* = 0.036), the incidence of PPCs (OR 13, 95% CI 3.2–52, *p* = 0.005), and continued mechanical ventilation postoperatively (OR 8.4, 95% CI 2–34, *p* < 0.001) as risk factors for in-hospital mortality (Figure 5).

## 4. Discussion

The results of this retrospective study of 152 patients undergoing pneumonectomy presents the latest data from a multicenter database, the German Thoracic registry, with an observed 21% incidence of PPCs and an associated mean mortality of 25% in the PPC group (compared to 3% in the non-PPC group). In this cohort, in order to identify high-risk patients, our definition of PPC included severe respiratory complications, which might explain the varying incidence and effects reported from other studies [4,9,10,11]. The results of our study revealed the following factors to be independently associated with PPCs, according to the multivariate analysis: a predicted preoperative FEV_1_ < 50%, an ICU stay of more than 2 days, and the occurrence of severe medical complications during the stay.

The impact of impaired lung function, particularly in patients with chronic obstructive pulmonary disease (COPD), on lung resection surgery is well documented [12,13], although spirometric values do not necessarily correspond to the postoperative residual values. The general debate regarding the limits of functional operability is ongoing and fueled by the heralding of minimally invasive surgery [14], which is usually not the standard approach for pneumonectomy. We could identify an increased risk for PPCs and in-hospital mortality in marginal patients, as well as significant mortality differences for the mean FEV_1_ and DLCO values. On a purely mathematical basis, patients with a predicted preoperative FEV_1_ < 50% have a 44% chance of experiencing PPCs and of not being discharged alive. Despite the discussed limitations [15], spirometric parameters can predict postoperative complications after pneumonectomy, either by identifying marginal patients or in a multimodality setting [16,17], triggering intensified perioperative respiratory care for these patients.

The primary postoperative care in the ICU or HDU is influenced by the individual structure and capacity of each hospital [18]. Therefore, in a multicenter study, the location of postoperative care may vary. The prolonged need for ICU care in the PPC group itself does imply a higher (co)incidence of complications, but not a causal relationship [19]. The increased rate might be due to preoperative factors not completely available from our data but may also result from a complicative intraoperative course, resulting in an impaired recovery and increased susceptibility to further complications, which is confirmed by our, and other, data [20].

The link between PPCs and medical complications (see Appendix A) might also indicate an interrelationship, e.g., new onset atrial fibrillation inducing cardiac congestion can result in respiratory failure or could be induced by the septic sequelae of postoperative pneumonia. No further discrimination, either in a causal or timely respect, is possible from this registry data.

No independent anesthesiologic factors could be identified. After the univariate analysis, higher intraoperative FiO_2_, driving pressure, and average inspiratory pressure during OLV were observed in patients with PPCs. Whether more invasive ventilatory settings and higher intraoperative oxygen demand reflect the effects of preexisting structural lung disease [21], or whether it may contribute to ventilator-induced injury, remains unclear in our study and would require further investigation considering the complexity of ventilator-induced injury in thoracic surgery [22,23]. If invasive ventilation remains, despite trials of optimization, it could identify patients at risk and trigger necessary perioperative measures, such as admission to intensive care, or intensified chest and general physiotherapy.

No significant differences were found between the regional anesthetic techniques in terms of their use, specifically in regards to continuous epidural or paravertebral anesthesia. While older studies report a benefit of epidural anesthesia, more recent data report equivocal effects or even increased complications in patients undergoing epidural anesthesia (compared to paravertebral techniques) [24]. A total of 72% of all patients received continuous neuraxial anesthesia. Among them, 88% were classified as ASA 3 or higher (vs. 71% in the single shot group, *p* = 0.025), and the duration of surgery (194 ± 78 vs. 166 ± 75 min, *p* = 0.04) was significantly longer, both representing relevant risk factors [25,26]. Considering the widespread use of neuraxial techniques in general, and their even more frequent use in diseased patients, an undetected (selection) bias cannot be excluded. Neuraxial anesthesia remains a trusted technique to provide optimized analgesia and effective, adapted, and multimodal analgesic techniques represents a mainstay of patient-centered anesthesia care.

Pulmonary hyperperfusion and fluid overload have been shown to be causative of PPCs in thoracic surgery and pneumonectomy patients [22,27,28]. The concept of restrictive fluid management in lung resection surgery is well established, although the thresholds are not clear, and postresection lung injury is not limited to the intraoperative fluid volume alone [29,30,31,32,33,34,35].

Considering the moderate rather than restrictive intraoperative net fluid balance, e.g., calculated for a patient with 80 kg body weight (mean operative duration x mean net balance) as 1257 mL (without PPC) vs. 1178 mL (with PPC) in both groups, no significant difference was found in our study. Fluid management remains an important topic in thoracic surgery, and goal directed management could be beneficial in avoiding hypo- and hypervolemia.

Surgical factors could not be identified as independent risk factors for PPCs. Patients with PPCs experienced significantly longer surgeries and higher rates of extensive resection involving the chest wall, as well as higher rates of reoperation, according to the univariate analysis, which is consistent with the results in the existing literature [26,34]. The underlying diagnosis did not result in any differences, but the small group of patients (22/152) undergoing surgery for issues other than bronchial carcinoma might not allow for further differentiation.

The independent risk factors for in-hospital death were as follows: incidence of PPCs, continuation of mechanical ventilation during the postoperative period, and the combination of a preoperative pulmonary infection (<4 weeks before surgery) and a preoperative FEV_1_ below 60% (Figure 5). Preoperative risk factors described in other studies, such as higher BMI, age, and ASA-scores, did not vary between the groups. Non-survivors exhibited higher rates of impaired lung function and neoadjuvant therapy in our study [35,36]. Intraoperative differences in non-survivors included higher blood loss, more allogenic transfusions, and a longer duration of surgery as indicators of more complicated surgery (together with the increased rate of reoperation and need for postoperative chest drain). In our study of 152 patients, the statistics are certainly influenced by the sample size, and these items reached a degree of significance only in the univariate analysis. Nevertheless, recognizing the clinical impact of these factors mentioned, i.e., prehabilitation and optimization of marginal patients, the prevention of hemorrhage, and optimized management, could contribute to better outcomes. As mentioned previously, the overall in-hospital mortality of patients with PPCs was 25%, regardless of the number of PPCs (Figure 3), compared to an in-hospital mortality of 2.5% in patients without PPCs. The overall in-hospital mortality in our study was 7.2%, which is within the range reported in recent studies [37,38]. In our study, 73% of the non-survivors exhibited at least one PPC, and the mortality correlated to the count of PPCs. Varying definitions exist within the general literature, also including minor complications, such as radiographic atelectasis, and those not necessarily related to primary perioperative pulmonary injury, such as pulmonary aspiration. The clinically based definitions chosen in our study identified high-risk patients and provided a correlation between the count of PPCs and the deleterious effects on mortality.

In our study, only one of seven patients ventilated for more than 48 h presented with no PPC or medical complications, whereas the remaining 6 (86%) had at least one PPC and at least one medical complication. The survival rate of patients who were extubated in the operation theater was 94%, and more than 30% of patients ventilated after the operation died during the hospital stay, a rate approaching the risk reported for unplanned reintubation [20]. As described in previous reports, continued mechanical ventilation itself may be associated with an increased risk of medical and pulmonary complications, which has been noted in other studies, although the rationale to continue postoperative ventilation is often based on several existing factors, including hypothermia, cardiorespiratory instability, or inadequate analgesia, but not all of these can be modified [33,34]. Determining the exact causal relationship between factors leading to and resulting from prolonged mechanical ventilation remains difficult, and our retrospective analysis can offer only statistic associations. Therefore, it would be reasonable to recognize the mechanisms and minimize the preceding sequelae, if modifiable, and to minimize the period of mechanical ventilation as much as possible rather than to avoid postoperative ventilation at any cost. In any case, prolonged postoperative ventilation identifies patients at risk, as described above.

Performing thoracic surgery, and especially pneumonectomy, in marginal patients with a recent pulmonary infection is a high-risk proposition [39]. Preoperative infectious processes, such as poststenotic pneumonia or necrotizing tumors, may further influence the postoperative course, but these are not completely modifiable. The inflammatory component, as shown by increased levels of C-reactive protein, suggest a role for an optimal perioperative conditioning of the patients, including intensified physiotherapy, pharmacological therapy, pulmonary hygiene, adequate antimicrobial therapy, and optimized intra- and postoperative management.

## 5. Limitations

The retrospective nature of our study might limit the conclusions drawn from our work. Only incomplete datasets were available for preoperative factors such as comorbidities and tumor staging, which were therefore excluded from the analysis, and preoperative risk factors, such as the Charlson Comorbidity Index, are not available. The multicenter nature of this study, with four different participating centers, might imply some bias due to variable perioperative management, although this might reflect only small differences in management in our daily clinical practice. The possible (clinical) correlation between the risk factors identified from our data might cast doubts in regards to the true “independence” of the factors, suggesting a more hierarchical significance. The relatively small number of patients in our cohort might influence the statistical effects of our results, and fewer identified modifiable risk factors might reduce the conclusions drawn from our study. However, considering the decreasing number of pneumonectomies performed due to the high morbidity and mortality, our study represents the most recent data obtained from a reasonably large cohort within a short period of time [2,10,38].

## 6. Conclusions

Despite the limitations mentioned above, our study allows us to draw important conclusions based on the many findings derived from pre-, intra- and postoperative parameters. The role of PPCs in this recent cohort of pneumonectomy patients becomes clear, considering the high morbidity and mortality of patients with PPCs compared with unaffected patients, implying a high medical and socioeconomic burden. The identification of the independent risk factors revealed a role of impaired pulmonary function parameters, the association between prolonged ICU stays, and the occurrence of medical complications. In-hospital mortality was increased in patients with a combination of a preoperative predicted FEV_1_ <60% and a recent pulmonary infection, postoperative ventilation, and PPCs. Additional factors reported from our data could further indicate an elevated perioperative risk, and the recognition of these factors during the preoperative selection, intraoperative management, and postoperative care should influence the perioperative risk stratification and management.

Considering the retrospective nature of our study, further investigations could shed light on the hypotheses raised in our study.

## Figures and Tables

**Table 1:** Univariate analysis of patient-related and preoperative parameters leading to risk factors for PPCs in patients undergoing pneumonectomy. The data are presented as percentages and the number of patients (n) or mean ± standard deviation. PPC = postoperative pulmonary complication; BMI = body mass index; ASA PS = American Society of Anesthesiologists Physical Status. DLCO = diffusing capacity of the lungs for carbon monoxide; FEV_1_ = forced expiratory volume in 1 s; CRP = C-reactive protein; Hb = Hemoglobin.

	No PPC 79% (120)	PPC 21% (32)	*p*-Value
*** Patient characteristics ***			
Age (years)	63.6 ± 9.5	62.1 ± 10.5	0.83
BMI	25.6 ± 0.5	26.2 ± 6	0.9
ASA PS ≥ 3	83% (100)	84% (27)	0.9
Male gender	73% (87)	78% (25)	0.68
Female	27% (33)	22% (7)	0.63
Smoking	88% (103)	87% (28)	1
Current smoking	44% (51)	56% (18)	0.23
Cessated smoking	44% (52)	31% (10)	0.22
***Preoperative characteristics***			
FEV_1_ (% predicted)	73 ± 20	67 ± 23	0.18
FEV_1_ < 50% (predicted)	8% (9)	33% (7)	<0.001
DLCO (% predicted)	72 ± 21	72 ± 28	0.9
Preoperative pulmonary infection + FEV_1_ < 60% predicted	4% (4)	14% (4)	0.04
CRP (mg/dL)	67 ± 89	56 ± 88	0.6
Hemoglobin (g/dL)	12.4 ± 1.9	12.1 ± 2.1	0.4

**Table 2:** Univariate analysis of anesthesiological and surgical characteristics leading to risk factors for PPCs in patients undergoing pneumonectomy. The data are presented as the percentage of all patients and number of patients (n) or mean ± standard deviation. PPC = postoperative pulmonary complication; TIVA = total intravenous anesthesia; OLV = one lung-ventilation; FiO_2_ = fraction of inspired oxygen; PEEP = positive endexpiratoy pressure, PRBC = packed red blood cells, FFP = fresh frozen plasma.

	No PPC 79% (120)	PPC 21% (32)	*p*-Value
*** Anesthesiological characteristics ***			
TIVA	60% (72)	72% (23)	0.30
Epidural catheter	68% (82)	72% (23)	0.33
Paravertebral catheter	4% (5)	0	0.58
Continuous neuraxial anesthesia technique	72% (86)	75% (24)	0.82
* Intraoperative ventilatory settings during OLV *			
Duration OLV (min)	167 ± 82	203 ± 114	0.06
Average FiO_2_ during OLV	0.61 ± 0.11	0.69 ± 0.15	0.01
Mean inspiratory pressure (mbar)	20.4 ± 4.3	23.2 ± 3.9	0.03
PEEP (mbar)	5.9 ± 1.5	6.5 ± 1.9	0.16
Driving pressure (mbar)	14.1 ± 4.2	17.1 ± 3.9	0.034
Respiratory minute volume (L/min)	6.3 ± 4.6	5.6 ± 1.3	0.37
* Intraoperative fluid balance *			
Crystalloid infusion (mL)	2389 ± 1312	2982 ± 2037	0.15
Crystalloid infusion (mL/kg/h)	12.4 ± 10	10.9 ± 5.5	0.45
Colloid infusion (mL)	199 ± 385	442 ± 842	0.11
Urine output (mL/kg/h)	1.4 ± 2	1.1 ± 1	0.49
Blood loss (mL)	712 ± 975	912 ± 881	0.075
Intraoperative fluid balance (mL/kg/h)	5.3 ± 5.9	4.0 ± 4.7	0.43
Intraoperative transfusion	71% (15)	28% (6)	0.36
PRBC (mL)	50 ± 220	150 ± 450	0.29
*** Surgical characteristics ***			
Right pneumonectomy	39.2% (47)	43.8% (14)	0.51
Operative duration (min)	178 ± 68	221 ± 98	0.006
Operative duration > 180 min	71.8% (51)	28.2% (20)	0.049
Blood loss (mL)	712 ± 975	912 ± 881	0.075
* Extended resection *			
None	36.7% (44)	43.8% (14)	0.54
+ angioplasty	5.8% (7)	6.3% (2)	0.9
+ intrapericardial resection	56.7% (66)	50% (16)	0.55
+ chest wall resection	4.2% (5)	15.6% (5)	0.046
+ diaphragmal resection	4.2% (5)	12.5% (4)	0.09
+ Re-operation	6.7% (8)	31.3% (10)	<0.001
* Histology *			
Bronchial carcinoma (BC)	89% (107)	84% (27)	0.4
Metastasis	2% (2)	6% (2)	0.1
Other neoplasm	3% (4)	3% (1)	1
Infectious disease	3% (3)	3% (1)	0.6
Benign non-infectious disease	3% (4)	3% (1)	0.7

**Table 3:** Univariate comparison of postoperative parameters in patients with and without postoperative pulmonary complications undergoing pneumonectomy. The data are presented as percentage and the number of (n) or mean ± standard deviation. PPC = postoperative pulmonary complication; ICU = intensive care unit; HDU = high dependency unit; SAPS = simplified acute physiology score; FiO_2_ = fraction of inspired oxygen; PEEP = positive endexpiratory pressure.

	No PPC (120)	PPC (32)	*p*-Value
*** Postoperative data ***			
Postoperative transfer to ICU	42% (50)	47% (15)	0.69
Postoperative transfer to HDU	46% (55)	50% (16)	0.69
ICU stay (d)	1.9 ± 1.4	10.5 ± 12.5	<0.001
>2 d ICU	9% (11)	38% (12)	<0.001
Hospital stay (d)	10.8 ± 5	21.4 ± 20	<0.001
Death during inpatient stay	3% (3)	25% (8)	<0.001
ventilated admission to ICU	6% (7)	19% (6)	0.03
* Ventilatory parameters in mechanically ventilated patients *			
postoperative ventilation (hours)	2.6 ± 8	140 ± 223	<0.001
SAPS initial	24.4 ± 12	45 ± 16	0.06
FiO_2_	0.4 ± 0.07	0.65 ± 0.2	0.56
PEEP (mbar)	6.5 ± 1.3	7.3 ± 1	0.38
Inspiratory pressure (mbar)	20.5 ± 6.6	24.5 ± 2.1	0.47
Respiratory rate (per min)	17 ± 4.7	21 ± 4.9	0.8
PaCO_2_ (mmHg)	45 ± 6	46 ± 5	0.68
PaO_2_ (mmHg)	155 ± 95	146 ± 70	0.8
* Medical complications *			
Medical complications	12% (14)	47% (15)	<0.001
Renal failure	1% (1)	9% (3)	0.03
Gastrointestinal complications	2% (2)	6% (2)	0.2
Arrhythmia	8% (9)	22% (7)	0.027
Myocardial infarction	0	6% (2)	0.043
Cardiopulmonary resuscitation	2% (2)	16% (5)	0.02
Sepsis	0.8% (1)	9% (3)	0.03

**Table 4:** Univariate analysis of patient and preoperative characteristics leading to risk factors for in-hospital death in patients undergoing pneumonectomy. The data are presented as percentage and the number of patients (n) or mean ± standard deviation. MC = medical complication; BMI = body mass index; ASA PS = American Society of Anesthesiologists Physical Status; FEV_1_ = forced expiratory volume in 1 s (preoperative, % predicted); DLCO = diffusing capacity of the lungs for carbon monoxide (preoperative % predicted); CRP = C-reactive protein; Hb = Hemoglobin.

	Survived (n = 141)	Died (n = 11)	*p*-Value
*** Patient characteristics ***			
Age (years)	62.5 ± 10.3	60.7 ± 5.4	0.56
BMI	25.7 ± 5.4	24.4 ± 6.6	0.44
Male gender	74% (108)	73% (8)	1
ASA PS ≥ 3	83% (117)	91% (10)	0.7
Smoking history	88% (121)	91% (10)	0.84
Respiratory infection < 4 weeks	14% (18)	44% (4)	0.04
*** Preoperative characteristics ***			
FEV_1_ (% predicted)	73 ± 20	58 ± 25	0.037
FEV_1_ < 50% predicted	6% (9)	64% (7)	<0.001
DLCO (% predicted)	73 ± 21.4	73 ± 21	0.037
DLCO < 50% predicted	13% (15)	55% (6)	0.006
Preoperative resp. Infection + FEV1 < 60% predicted	3% (4)	40% (4)	<0.001
CRP (mg/mL)	56 ± 67	114 ± 145	0.046

**Table 5:** Univariate analysis of anesthesiologic and surgical data leading to risk factors for in-hospital mortality in patients undergoing pneumonectomy. The data are presented as percentage and the number of patients (n) or mean ± standard deviation. MC = medical complication; TIVA = total intravenous anesthesia; OLV = one lung-ventilation; FiO_2_ = fraction of inspired oxygen; PEEP = positive endexspiratory pressure, PRBC = packed red blood cells, FFP = fresh frozen plasma; BSI = bronchial stump insufficiency.

	Survived 93% (n = 141)	Died 7% (n = 11)	*p*-Value
*** Anesthesiological characteristics ***			
TIVA	62% (88)	64% (7)	0.14
Epidural catheter	70% (98)	82% (9)	0.51
Paravertebral catheter	3% (4)	9% (1)	0.37
Continuous neuraxial anesthetic technique	72% (101)	82% (9)	0.73
* Intraoperative ventilatory settings during OLV *			
Average PEEP (mbar)	6.4 ± 1.6	6.6 ± 2.2	0.44
Driving pressure (mbar)	14.7 ± 4.3	11.7 ± 4.5	0.22
OLV duration (min)	172 ± 86	206 ± 132	0.56
OLV > 180 min + FEV1 < 60% predicted	7% (9)	27% (3)	0.007
* Intraoperative fluid management *			
Crystalloid infusion (mL)	2406 ± 1304	3537 ± 2925	0.015
Crystalloid mL/kg/h	12.1 ± 9.4	10.8 ± 5.8	0.65
Colloid infusion (mL)	231 ± 484	400 ± 651	0.464
Net infusion volume (mL/kg/h)	4.9 ± 5.7	5.3 ± 5.5	0.84
Urine volume (mL/kg/h)	1.3 ± 1.8	2.1 ± 2.2	0.19
Blood loss (mL)	631 ± 545	2059 ± 2493	<0.001
Blood loss > 1500 mL	6% (7)	36% (4)	0.007
PRBC (mL)	72 ± 240	1092 ± 2200	<0.001
*** Surgical characteristics ***			
Duration of surgery (min)	178 ± 68	221 ± 98	0.006
+ Re-operation	10% (14)	36% (4)	0.027
Postoperative Drainage (d)	2.2 ± 3.5	14.5 ± 12	<0.001
BSI	5% (7)	27% (3)	0.026
* Histology *			
Bronchial carcinoma	93% (125)	82% (9)	0.7
Metastasis	3% (4)	0%	0.3
Other neoplasm	3% (4)	9% (1)	0.8
Infectious disease	2% (3)	9% (1)	0.7
Benign non-infectious disease	4% (5)	0	0.2

**Table 6:** Univariate analysis of patient and preoperative characteristics leading to risk factors for in-hospital death in patients undergoing pneumonectomy. The data are presented as percentage and the number of patients (n) or mean ± standard deviation. ICU = intensive care unit; HDU = high dependency unit; SAPS = simplified acute physiology score; PPC = postoperative pulmonary complications.

	Survived 93% (n = 141)	Died 7% (n = 11)	*p*-Value
*** Postoperative characteristics ***			
Primary ICU admission	43% (60)	46% (5)	0.32
Primary HDU admission	55% (78)	46% (5)	1
ICU stay (d)	3.6 ± 6	16.8 ± 22	<0.001
Postoperative hospital stay (d)	10.8 ± 5	21.4 ± 19.8	<0.001
admitted to the ICU ventilated	6% (9)	31% (4)	0.008
Duration of ventilation (hours)	28 ± 105	302 ± 467	<0.001
SAPS on admission day	27 ± 14	32	0.8
* Postoperative complications *			
PPCs	17% (24)	73% (8)	<0.001
Medical complications	16% (23)	55% (6)	0.007
Surgical complications	18% (25)	55% (6)	0.01

## Data Availability

Data are contained within the article.

## Supplementary Materials

The following supporting information can be downloaded at: https://www.mdpi.com/article/10.3390/jcm13010035/s1, Table S1: Strobe Document.

## Appendix A

**Table A1:** Univariate analysis of patient characteristics leading to risk factors for medical complications in patients undergoing pneumonectomy. The data are presented as the percentage of all patients and number (n) or mean ± standard deviation. MC = medical complication; BMI = body mass index; ASA PS = American Society of Anesthesiologists Physical Status.

	No MC 80% (n = 123)	MC 20% (n = 29)	*p*-Value
*** Patient characteristics ***			
Age (years)	62.1 ± 10	63.8 ± 10	0.37
Male	75% (92)	69% (20)	0.56
Female	25% (31)	31% (9)	0.64
BMI	27 ± 4	28.4 ± 5.2	0.12
BMI > 30	16% (20)	34% (10)	0.037
ASA PS ≥ 3	81% (105)	76% (22)	0.26
Active smoking	52% (64)	17% (5)	0.77
Cessated smoking	46% (57)	17% (5)	0.09
Never smoked	11% (14)	14% (4)	0.31
Respiratory infection < 4 weeks	15% (18)	14% (4)	0.04

**Table A2:** Univariate analysis of preoperative parameters leading to risk factors for medical complications in patients undergoing pneumonectomy. The data are presented as the percentage of all patients and number (n) or mean ± standard deviation. MC = medical complication; FEV_1_ = forced expiratory volume in 1 s (preoperative (%) predicted); DLCO = diffusing capacity of the lungs for carbon monoxide (preoperative (%) predicted); CRP = C-reactive protein; Hb = Hemoglobin.

	No MC 80% (n = 123)	MC 20% (n = 29)	*p*-Value
*** Preoperative characteristics ***			
FEV1 (% predicted)	73.5 ± 20	62.4 ±18.6	0.037
FEV1 < 50% predicted	10% (12)	21% (6)	0.09
DLCO (% predicted)	74 ± 22.4	63.2 ± 20.7	0.04
DLCO < 50% predicted	11% (13)	24% (7)	0.04
PaO_2_ (mmHg) preoperative	73 ± 8.6	70 ± 9.4	0.12
PaCO_2_ (mmHg) preoperative	37 ± 3.2	37 ± 5.7	0.41
CRP (mg/dL)	58.3 ± 69	74.7 ± 106	0.4
Hemoglobin (g/dL)	12.4 ± 1.9	12.3 ± 2.1	0.81
Hb < 10 g/dL	11% (13)	17% (5)	0.34
Leucocytes (10^4^/dL)	10.4 ± 4.4	10 ± 3.3	0.62
Previous lung surgery	6% (7)	14% (4)	0.21
Previous radiation	5% (6)	14% (4)	0.09
Preoperative chemotherapy	13% (16)	17% (5)	0.53
No preoperative therapy	44% (54)	34% (10)	0.29

**Table A3:** Univariate analysis of anesthesiologic data leading to risk factors for medical complications in patients undergoing pneumonectomy. The data are presented as percentage and the number of patients (n) or mean ± standard deviation. MC = medical complication; TIVA = total intravenous anesthesia; OLV = one lung-ventilation; FiO_2_ = fraction of inspired oxygen; PEEP = positive endexpiratoy pressure, PRBC = packed red blood cells, FFP = fresh frozen plasma.

	No MC 80% (n = 123)	MC 20% (n = 29)	*p*-Value
*** Anesthesiological characteristics ***			
Volatile Anesthetics	38% (47)	34% (10)	0.45
TIVA	84% (103)	90% (26)	0.57
Epidural catheter	69% (85)	76% (22)	0.65
Paravertebral catheter	3% (4)	3% (1)	1
Continuous neuraxial anesthetic technique	72% (88)	76% (22)	0.8
Paravertebral single shot anesthesia	7% (9)	3% (1)	0.68
CPAP non-ventilated lung	5% (6)	0	0.34
* Intraoperative ventilatory settings during OLV *			
Mean inspiratory pressure (mbar)	20.9 ± 4.2	21 ± 5.1	0.9
PEEP (mbar)	6 ± 1.6	5.8 ± 1.6	0.8
Driving pressure (mbar)	14.5 ± 4.1	15 ± 5.2	0.76
Mean intraoperative FiO_2_	0.63 ± 0.11	0.65 ± 0.18	0.56
Lowest intraoperative FiO_2_	0.55 ± 0.12	0.57 ± 0.16	0.8
Recruitment maneuvres performed	28% (34)	24% (7)	0.63
* Intraoperative fluid management *			
Crystalloid infusion (mL)	2409 ± 1292	2822 ± 2147	0.52
Crystalloid (mL/kg/h)	12.4 ± 9.7	10.5 ± 6.1	0.5
Colloid infusion (mL)	220 ± 386	339 ± 829	0.61
Urine output (mL)	364 ± 433	420 ± 312	0.12
Urine volume (mL/kg/h)	1.3 ± 1.9	1.6 ± 1.5	0.09
Blood loss (mL)	663 ± 657	1087 ± 1636	0.38
Blood loss > 1000 mL	15% (19)	28% (8)	0.19
Blood loss > 2000 mL	2% (3)	14% (4)	0.032
PRBC (mL)	84 ± 261	414 ± 1320	0.017
FFP (mL)	37 ± 275	137 ± 387.5	0.16
Platelet concentrate (mL)	0	20 ± 32	0.03

**Table A4:** Univariate analysis of surgical characteristics leading to risk factors for medical complications in patients undergoing pneumonectomy. The data are presented as percentage and the number of patients (n) or mean ± standard deviation. MC = medical complication.

	No MC 80% (n = 123)	MC 20% (n = 29)	*p*-Value
*** Surgical characteristics ***			
Emergency procedure	7% (9)	7% (2)	1
Operative duration (min)	179 ± 68	216 ± 109	0.14
Operative duration > 180 min	46% (57)	48% (14)	1
Operative duration > 300 min	57% (8)	21% (6)	0.03
Blood loss (mL)	663 ± 657	1087 ± 1636	0.36
* Extended surgery *			
None	35% (43)	52% (15)	0.14
+ Angioplasty	7% (8)	3% (1)	1
+ Pericardial resection	58% (71)	45% (13)	0.22
+ Chest wall resection	6% (7)	17% (5)	0.006
+ Diaphragmatic resection	3% (4)	17% (5)	0.013
Re-Operation	9% (11)	24% (7)	0.048
Postoperative Drainage (d)	1.9 ± 1.9	5.7 ± 9.4	<0.001
Postoperative transfusion	5% (6)	10% (3)	0.37

**Table A5:** Univariate analysis of postoperative characteristics leading to risk factors for medical complications in patients undergoing pneumonectomy. The data are presented as percentage and the number of patients (n) or mean ± standard deviation. MC = medical complications; ICU = intensive care unit; HDU = high dependency unit; SAPS = simplified acute physiology score; FiO_2_ = fraction of inspired oxygen; PEEP = positive endexpiratory pressure; PPC = postoperative pulmonary complications.

	No MC 90% (n = 123)	MC 20% (n = 29)	*p*-Value
*** postoperative characteristics ***			
Primary ICU admission	40% (49)	55% (16)	0.15
ICU stay (d)	2.1 ± 1.6	12.3 ± 15	<0.001
SAPS on admission	12.7 ± 4.5 (8)	33.2 ± 7.1 (5)	0.32
>2 d ICU	52% (12)	38% (11)	0.002
>5 d ICU	2% (3)	21% (6)	0.005
Primary HDU admission	57% (70)	45% (13)	0.3
Postoperative hospital stay (d)	11.8 ± 9.7	19.8 ± 13.5	<0.001
Ventilated admission to ICU	5% (6)	24% (7)	0.003
* Ventilatory parameters in mechanically ventilated patients *			
Hours ventilated	2.6 ± 8.1	194 ± 309	0.02
Ventilated > 48 h	1% (1)	21% (6)	<0.001
FiO_2_	0.41 ± 0.09	0.56 ± 0.21	0.22
PEEP (mbar)	6.5 ± 1.3	7 ± 1.4	0.87
Average inspiratory pressure (mbar)	18 ± 5.3	25.6 ± 2.5	0.08
Respiratory rate (per min)	14.6 ± 2.3	18.7 ± 4.4	0.21
PaCO_2_ (mmHg)	44.8 ± 6	44.7 ± 6.5	0.97
PaO_2_ (mmHg)	157 ± 96	139 ± 67	0.96
* Postoperative complications *			
PPC	14% (17)	52% (15)	0.001
Deceased	4% (5)	21% (6)	0.007
Number PPC	0.2 ± 0.45	1.1 ± 1.27	<0.001
Surgical complications	20% (25)	21% (6)	1

**Table A6:** Results from the multivariate analysis identifying risk factors for medical complications in patients undergoing pneumonectomy. The Odds Ratio (OR) and the 95% confidence interval (CI) are shown. ICU = Intensive care unit; PPC = postoperative pulmonary complication.

Risk Factors for Medical Complications			
	*p*-Value	OR	95% CI
admitted to the ICU ventilated	0.028	4.4	1.2–17
PPC	<0.001	5.5	2.1–14.7
