# Supplementary material for: The Impact of Postoperative Pulmonary Complications on Perioperative Outcomes in Patients Undergoing Pneumonectomy: A Multicenter Retrospective Cohort Study of the German Thorax Registry
Source: J Clin Med. 2023 Dec 20;13(1):35. doi: 10.3390/jcm13010035 (PMC10779566; doi:10.3390/jcm13010035)
Supplement: Supplementary file 1 [file jcm-13-00035-s001.zip › jcm-2721757-supplementary.pdf]

STROBE Statement—Checklist of items that should be included in reports of ***cohort studies***

	**Item No**	**Recommendation**	**Page No**
**Title and abstract**	1	(*a*) Indicate the study's design with a commonly used term in the title or the abstract	See abstract
		(*b*) Provide in the abstract an informative and balanced summary of what was done and what was found	See abstract
**Introduction**			
Background/rationale	2	Explain the scientific background and rationale for the investigation being reported	See introduction Page 1
Objectives	3	State specific objectives, including any prespecified hypotheses	See introduction Page 1
**Methods**			
Study design	4	Present key elements of study design early in the paper	See Material and Methods
Setting	5	Describe the setting, locations, and relevant dates, including periods of recruitment, exposure, follow-up, and data collection	See Material and Methods
Participants	6	(*a*) Give the eligibility criteria, and the sources and methods of selection of participants. Describe methods of follow-up	See Material and Methods and Figure 1
		(*b*) For matched studies, give matching criteria and number of exposed and unexposed	
Variables	7	Clearly define all outcomes, exposures, predictors, potential confounders, and effect modifiers. Give diagnostic criteria, if applicable	See Methods section/ references
Data sources/ measurement	8*	For each variable of interest, give sources of data and details of methods of assessment (measurement). Describe comparability of assessment methods if there is more than one group	See Material & Methods
Bias	9	Describe any efforts to address potential sources of bias	
Study size	10	Explain how the study size was arrived at	
Quantitative variables	11	Explain how quantitative variables were handled in the analyses. If applicable, describe which groupings were chosen and why	
Statistical methods	12	(*a*) Describe all statistical methods, including those used to control for confounding<br>(*b*) Describe any methods used to examine subgroups and interactions<br>(*c*) Explain how missing data were addressed<br>(*d*) If applicable, explain how loss to follow-up was addressed<br>(*e*) Describe any sensitivity analyses	See statistics
**Results**			

Participants	13*	(a) Report numbers of individuals at each stage of study—eg numbers potentially eligible, examined for eligibility, confirmed eligible, included in the study, completing follow-up, and analysed<br>(b) Give reasons for non-participation at each stage<br>(c) Consider use of a flow diagram	See figure 1<br><br><br>See figure 1
Descriptive data	14*	(a) Give characteristics of study participants (eg demographic, clinical, social) and information on exposures and potential confounders<br>(b) Indicate number of participants with missing data for each variable of interest<br>(c) Summarise follow-up time (eg, average and total amount)	See results section
Outcome data	15*	Report numbers of outcome events or summary measures over time	See results section

Main results	16	(*a*) Give unadjusted estimates and, if applicable, confounder-adjusted estimates and their precision (eg, 95% confidence interval). Make clear which confounders were adjusted for and why they were included<br>(*b*) Report category boundaries when continuous variables were categorized<br>(*c*) If relevant, consider translating estimates of relative risk into absolute risk for a meaningful time period	See results section
Other analyses	17	Report other analyses done—eg analyses of subgroups and interactions, and sensitivity analyses	See results section
**Discussion**			
Key results	18	Summarise key results with reference to study objectives	See discussion section
Limitations	19	Discuss limitations of the study, taking into account sources of potential bias or imprecision. Discuss both direction and magnitude of any potential bias	See limitations section
Interpretation	20	Give a cautious overall interpretation of results considering objectives, limitations, multiplicity of analyses, results from similar studies, and other relevant evidence	See discussion
Generalisability	21	Discuss the generalisability (external validity) of the study results	See limitations/ conclusions
**Other information**			
Funding	22	Give the source of funding and the role of the funders for the present study and, if applicable, for the original study on which the present article is based	There is no funding source apart from institutional funding

*Give information separately for exposed and unexposed groups.

Note: An Explanation and Elaboration article discusses each checklist item and gives methodological background and published examples of transparent reporting. The STROBE checklist is best used in conjunction with this article (freely available on the Web sites of PLoS Medicine at http://www.plosmedicine.org/, Annals of Internal Medicine at http://www.annals.org/, and Epidemiology at http://www.epidem.com/). Information on the STROBE Initiative is available at http://www.strobe-statement.org.
